# Delayed Presentation With Atypical Extrathyroidal Manifestations of Sporadic Non-autoimmune Congenital Hyperthyroidism: A Case Report and Literature Review

**DOI:** 10.7759/cureus.91040

**Published:** 2025-08-26

**Authors:** Rushikesh Dighe, Veenu Jain, Anshita Aggarwal, Bindu Kulshreshtha

**Affiliations:** 1 Endocrinology, Dr. Ram Manohar Lohia Hospital and Post Graduate Institute of Medical Education and Research, New Delhi, IND

**Keywords:** brachymetatarsia, congenital hyperthyroidism, dysmorphic fearures, mitral valve prolapse, pediatric thyrotoxicosis, radioiodine ablation, sporadic non-autoimmune, sporadic non-autoimmune congenital hyperthyroidism, tsh receptor mutation

## Abstract

Sporadic non-autoimmune congenital hyperthyroidism (SNAH) is a rare form of persistent thyrotoxicosis caused by germline activating mutations in the thyroid-stimulating hormone (TSH) receptor (TSHR) gene, distinct from the more common autoimmune neonatal hyperthyroidism. SNAH typically presents early with variable severity but often lacks the overt autoimmune features, leading to diagnostic delays. Extrathyroidal manifestations remain underrecognized in the sporadic form.

We report a case of SNAH from India in a 15-year-old male with a heterozygous activating mutation in exon 10 of the TSHR gene: p.Asp633Glu, caused by a de novo pathogenic nucleotide variant (c.1899C>G). To the best of our knowledge, this is the first documented case of SNAH from the Indian subcontinent. The patient initially presented at seven years of age with mild thyrotoxic features, dysmorphic facies (ocular telecanthus, flat nasal bridge), bilateral brachydactyly (short 3rd-5th metacarpals/metatarsals), and mitral valve prolapse. While some of such extra-thyroidal phenotypic features have been previously reported in familial non-autoimmune hyperthyroidism (FNAH), particularly those associated with TSHR mutation syndrome, they have not been described in any published case of sporadic non-autoimmune congenital hyperthyroidism, thereby expanding the phenotypic spectrum of SNAH. Despite initial biochemical control with carbimazole, the patient experienced disease progression requiring two radioiodine ablations. This case uniquely demonstrated delayed onset, persistently uncontrolled disease, delayed bone age despite thyrotoxicosis, and extra-thyroidal features as described above novel to SNAH.

This report expands the phenotypic spectrum of SNAH, highlighting atypical extrathyroidal manifestations such as facial dysmorphism and skeletal abnormalities. The identified Asp633Glu mutation has previously been reported in toxic adenomas as well as in one case of SNAH; however, the pathologic variant (c.1899C>G) is newly documented in this case report. A comprehensive review of 19 published SNAH cases emphasizes the clinical heterogeneity and lack of consistent genotype-phenotype correlation. Our case reinforces the importance of early genetic testing in persistent, antibody-negative thyrotoxicosis and underscores the role of definitive therapy in severe or refractory disease. Additionally, while most reported cases of SNAH present in the neonatal period or early infancy, this case was notable for its delayed presentation at seven years of age, further contributing to its diagnostic uniqueness.

This is the first Indian case of genetically confirmed SNAH associated with the Asp633Glu TSHR mutation, presenting with previously undescribed extrathyroidal features (e.g., brachydactyly, ocular telecanthus, flat nasal bridge, mitral valve prolapse). The delayed clinical onset in this case, compared to the typically early presentation in SNAH, underscores the importance of considering this diagnosis even beyond infancy in cases of persistent thyrotoxicosis. Early recognition of atypical phenotypes and consideration of SNAH in differential diagnosis can prompt timely intervention, improving developmental and metabolic outcomes.

## Introduction

Sporadic non-autoimmune congenital hyperthyroidism (SNAH) is a rare and persistent form of neonatal thyrotoxicosis caused by activating mutations in the thyroid-stimulating hormone receptor (TSHR) gene. Unlike the more commonly encountered neonatal thyrotoxicosis due to transplacental passage of maternal TSH receptor antibodies in Graves’ disease, SNAH occurs independently of maternal autoimmune status and lacks spontaneous remission [1]. The clinical presentation is highly variable, and almost all reported cases were found to have symptomatic thyrotoxicosis of varying severity within the first year of life with features such as tachycardia, failure to thrive, developmental delays, and cardiac complications [2]. While diverse phenotypic changes, including craniofacial dysmorphism and skeletal abnormalities, are well-documented in familial forms of congenital hyperthyroidism, they are rarely reported in sporadic cases, which may lead to diagnostic delays [3]. This case highlights the complex clinical course of a child with SNAH who presented with both typical thyrotoxic features with an unusual delay in onset and previously underreported extrathyroidal manifestations (bilateral brachydactyly in all four limbs, ocular telecanthus, mitral valve prolapse), reinforcing the need for heightened awareness of the broader phenotypic spectrum associated with this rare disorder.

## Case presentation

**Figure 1:**
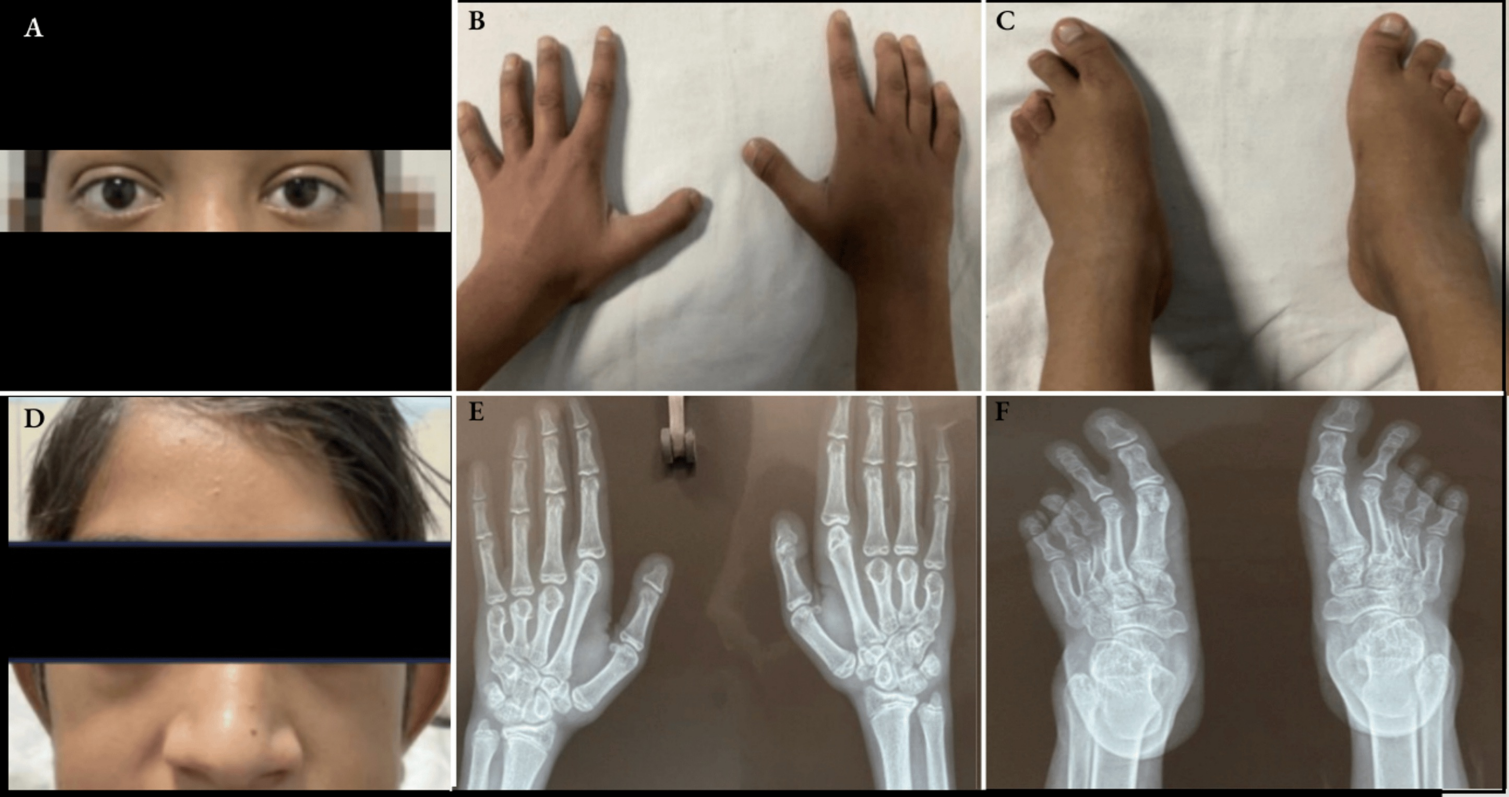
Dysmorphic and skeletal features in patient with SNAH (Panels A-F) A: Ocular telecanthus with positive Stellwag’s sign, B: Hand photograph (3rd-5th metacarpal shortening), C: Foot photograph (3rd-5th metatarsal shortening), D: Flat nasal bridge, E: Hand X-ray, F: Foot X-ray (Images have been anonymized in accordance with patient privacy guidelines). SNAH: Sporadic non-autoimmune congenital hyperthyroidism.

The index case is a 15-year-old male child with no family history of thyroid illness, who presented with features of thyrotoxicosis (tremors, decreased bowel transit time, New York Heart Association (NYHA) II dyspnoea) at seven years of age. Patient had antecedent history of preterm birth (34 weeks+2 days, birth weight 2400 grams) with delayed developmental milestones more affected in the cognitive and fine motor domains, while preserved gross motor and social domain (standing without support-12 months, pincer grasp-15 months, monosyllables-15 months). Thereafter, parents' recall of satisfactory growth and development compared to the child's age-equal peers. During his first presentation at seven years of age, he had average scholastic performance. He was brought to medical attention in view of the recent onset of shortness of breath, which had progressed rapidly over a span of 2 months (NYHA I -> III). At the first visit, his weight was 16 kgs (weight standard deviation score (WtSDS)-3.3), and his height was 116.5 (height standard deviation score (HtSDS)-2.1). Heart rate-122/min and blood pressure 98/50 mmHg. He had features of facial dysmorphism like ocular telecanthus (Figure 1A), flat nasal bridge (Figure 1D), vertically oblong skull with triangular facies, bilateral short 3rd/4th/5th metacarpal (Figures 1B, 1E), and short 3rd/4th/5th metatarsal (Figures 1C, 1F). On evaluation of dyspnoea, the patient was diagnosed to have mitral valve prolapse with severe mitral regurgitation and severe tricuspid regurgitation. He had a mild goitre, which previously had been unnoticed by his parents, and had no e/o exophthalmos. His free triiodothyronine (FT3) was 18.37 pg/ml (2-4.4 pg/ml) and free thyroxine (FT4) was 3.85 ng/dl (0.6-2.2 ng/dl), which were elevated, whereas he had suppressed TSH of 0.015 mIU/ml (0.5-5). His anti-TPO and TSH receptor auto-antibody titres were negative. Both of his parents were clinically and biochemically euthyroid with anti-tpo titres within the reference range. A radioactive iodine uptake (RAIU) scan was suggestive of diffuse thyroid uptake of 57.5% and 80.2% at two hours and 24 hours, respectively, consistent with hyper-functioning thyroid tissue. The patient did not have any features of McCune Albright Syndrome.

The patient was initiated on carbimazole at seven years of age. During eight years of follow-up and regular treatment, despite frequent up-titration of the dose of carbimazole, especially in the later five years, his biochemical control was rarely achieved (Table 1). However, the patient never had features of thyrotoxic crisis or overt hyperthyroidism during eight years of follow-up. His pubertal stages were consistent with chronological age, and he entered puberty at 13 years of age. Height SDS continued to be in -1 to -2 SDS, whereas weight SDS was affected the worst (-3 to -4 SDS) throughout the years of follow-up. Bone age remained delayed throughout the years of follow-up. In view of persistent uncontrolled disease despite high doses of carbimazole, he subsequently received the first dose of radioiodine (RAI) ablation (I131-10mCi) at 15 years of age and was reinitiated on carbimazole one month post ablation. However, in view of a persistent thyrotoxic state and no reduction in the dose of carbimazole, the patient was subjected to a second dose of RAI (I131-15mCi) six months after the first dose.

**Table 1:** Trend of thyroid function tests and developmental clinical endpoints in the patient Reference ranges for biochemical parameters: Free triiodothyronine (FT3) (2.0–4.4 pg/mL), free thyroxine (FT4) (0.6–2.2 ng/dL), thyroid-stimulating hormone (TSH)(0.5–5.0 µIU/mL). Height (Ht) standard deviation score (SDS) and Weight (Wt) standard deviation score (SDS) normal range: –2 to +2. Height velocity varies with pubertal stage - 5–7 cm/year in prepubertal children, 6–10 cm/year during pubertal growth spurt, and 4–8 cm/year in late puberty. Bone age was interpreted against expected chronological age ±1 year. Testicular volume was assessed based on pubertal staging: <4 cc (prepubertal), 4–10 cc (early puberty), and 10–15 cc (late puberty). “N/A” indicates data not available or not applicable. All laboratory values have been converted into standard clinical units for consistency.

Age	1st presentation (age 7 years)	11 years	13 years	15 years	Post 1st RAI Ablation	Post 2nd RAI Ablation (3 months interval)
FT3 (2-4.4 pg/ml)	5.05	3.73	14	12.90	5.44	0.92
FT4 (0.6-2.2 ng/dl)	2.03	0.77	4.7	6.35	3.2	0.24
TSH (0.5-5 mIU/ml)	0.009	0.001	0.01	0.02	0.01	3.118
Ht SDS (Normal: -2 to +2)	-2.1	-1.9	-1.82	-1.63	N/A	N/A
Height Velocity (Normal HV Range)	4 cm / year (5-7 cm/year)	3.7 cm / year (4-6 cm/year)	6.6 cm/year (6-10 cm/year)	7.2 cm / year (4-8 cm/year)	N/A	N/A
Wt SDS (Normal: -2 to +2)	-3.3	-3.2	-3.90	-3.7	N/A	N/A
Bone Age (Expected: Chronological Age +/- 1 year)	5.5 years	9.5 years	11 to 12 years	14 years	N/A	N/A
Pubertal onset: Testicular volume [Expected range (pubertal status)]	Rt - 2 cc Lt - 2 cc [1-3 cc (prepubertal)]	Rt - 2.5 cc Lt - 3.0 cc [3-4 cc (early pubertal)]	Rt - 4.0cc Lt - 4.5cc [4-10 cc (progressive puberty)]	Rt – 14 cc Lt – 10 cc [10-15cc (late puberty)]	N/A	N/A
Carbimazole Dose (mg per day)	15	12.5	40 mg	45 mg	30 mg	Hypothyroidism, initiated on levothyroxine 50 ug OD

**Figure 2:**
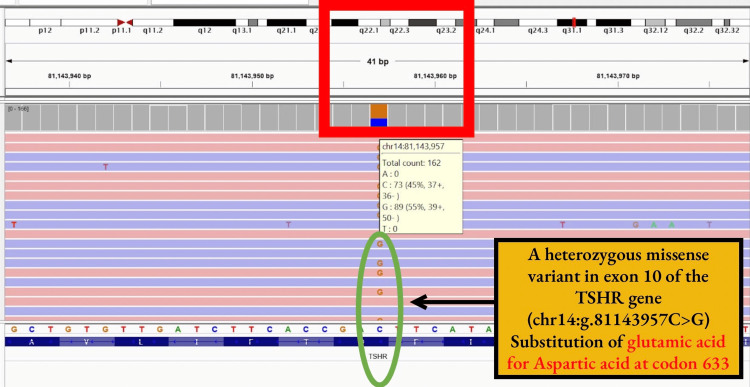
Whole Exome Sequencing The image is showing heterozygous missense variant in exon 10 of the TSHR gene (chromosome.14:g.81143957C>G).

Whole Exome Sequencing (WES) with an average sequencing depth (X) of 268 and average on-target sequencing depth (X) of 105.78 and high confidence variation detection (98.7% of target bases covered at >20X) was performed after obtaining parental consent, using genomic DNA extracted from a single EDTA-drawn peripheral blood sample of the index case. Library preparation was done using a custom gene capture kit, and sequences obtained were aligned to the human reference genome (GRCh38) using the BWA aligner on the Illumina NovaSeq 6000 platform to a mean exome-wide depth of > 80-100X and further to 160X after identification of the variant of interest (c.1899C>G) using Sentieon (GATK best practices framework). Selective capture and sequencing of the protein coding regions and clinically relevant genome was performed through which a heterozygous missense mutation in exon 10 of chr14. C1899C>G (p.Asp633Glu) was detected and reported as "Pathogenic" (ACMG) (Figure 2), resulting in the amino acid substitution of glutamic acid for aspartic acid at codon 633. The patient had post-ablation hypothyroidism after two doses six months apart and was initiated on levothyroxine in view of the development of new symptoms of hypothyroidism and currently is kept under close follow-up.

## Discussion

The TSH receptor (TSHR) belongs to the superfamily of seven transmembrane domain receptors coupled to G proteins. This gene is encoded by 10 exons, which span over 60kb on chromosome 14. Somatic activating mutations either in TSHR or the Gs alpha proteins constitutively activate the cAMP cascade and induce growth and hyperfunction of thyroid follicular cells, thereby leading to thyroid autonomy [4]. These mutations may be inherited as autosomal dominant/familial NAH or can occur sporadic/de novo non-immune hyperthyroidism (SNAH). In 1995, Kopp et al. first reported a case that presented with features of thyrotoxicosis without autoantibodies or autoimmune stigmata in a neonate [5]. Later, Holzapfel et al. identified a new germline gain-of-function mutation at position 505 in the third transmembrane region in the TSH receptor, leading to constitutive activation of the cyclic adenosine monophosphate (cAMP) cascade [6]. To date, only 13 loci in 19 cases of sporadic NAH have been reported worldwide. All cases had a mutation in the transmembrane region except in one case, which had an extracellular domain affected [7]. The index case has a point mutation leading to a heterozygous missense variant in Exon 10 of the 6th transmembrane domain of the TSHR gene transcript that results in the amino acid substitution of Glutamic acid for aspartic acid at codon 633. Such point mutations at codon 633 have previously been identified in toxic adenomas as well as autonomous thyroid nodules in patients with hyperthyroidism [8]. The observed amino acid change from aspartate to glutamate due to alteration of one allele at codon 633 in the index case has previously also been reported in a South Korean child who presented with thyrotoxic features during the neonatal period [9]. However, a different transition point mutation of C>G, as well as a multitude of dysmorphic features as described earlier, are novel in this case. Due to financial constraints, genetic testing of the parents could not be performed; however, both parents were clinically and biochemically euthyroid and tested negative for anti-thyroid peroxidase antibodies, making a familial form of nonautoimmune congenital hyperthyroidism unlikely.

Table 1 summarizes the trend of thyroid function tests from initiation of therapy till the 2nd RAI Ablation. In the initial few years of therapy, the disease was mild, and activity was controlled with successful tapering of the dose to 12.5 mg once a day with thyroid function test (TFT) in the euthyroid range. However, the requirement escalated in subsequent years with failure to achieve euthyroid hormone levels even on 45 mg once a day. 

His weight was affected more severely than height, which further showed catch-up after achieving normal pubertal growth spurt for height, but continued to remain poor for weight. He achieved puberty at 13 years with nocturnal penile intumescence at 14.5 years. To our surprise, despite uncontrolled hyperthyroid states, bone age was never advanced and, in fact, it lagged behind the chronological age with an average delay of six months. One plausible explanation for this observation could be persistently elevated metabolic demand with proportionately insufficient caloric intake, thereby delaying skeletal maturation. An alternative consideration is partial growth hormone deficiency secondary to chronic thyrotoxicosis, a phenomenon previously described in the literature [10]. However, growth hormone (GH) stimulation testing was not performed in this patient, given the consistent observation of more severe impairment in weight SDS (worse than -3SD) compared to relatively preserved height SDS throughout follow-ups, which makes the former explanation more likely. 

Patient continued to require progressively increasing doses of antithyroid drug to maintain euthyroid states during the recent few years. He was thereby subjected to RAI (radioiodine) ablation with I131-10mCi at the age of 15 years, but continued to stay thyrotoxic post first ablation. Six months later, he was subjected to a second dose of RAI-131-15mCi. Following the second radioiodine ablation, our patient demonstrated biochemical hypothyroidism with progressively declining FT4 and FT3 levels but paradoxically normal TSH concentrations. Thyroxine replacement was initially deferred in view of the non-elevated TSH; however, with worsening biochemical hypothyroidism and overt clinical features, levothyroxine was subsequently initiated. This phenomenon has been described in the literature as “disproportional TSH hyposecretion” or a central hypothyroid pattern after radioiodine therapy in nonautoimmune hyperthyroidism [11,12]. The mechanism is not fully elucidated, but proposed explanations include impaired hypothalamic-pituitary-thyroid axis setpoint following long-standing untreated thyrotoxicosis, altered ultrashort-loop feedback regulation at the pituitary level, or persistent TSH suppression despite low thyroid hormone concentrations. These reports emphasize the importance of relying on thyroid hormone concentrations and clinical features rather than TSH alone for therapeutic decisions in such patients.

A systematic review by Lueblinghoff et al. (2010) analyzed 14 cases of sporadic nonautoimmune congenital hyperthyroidism (SNAH) caused by activating TSH receptor mutations [13]. Despite wide variability in clinical presentation and treatment outcomes, no consistent correlation was found between the mutation’s in vitro activity and disease severity. Interestingly, even patients with the same mutation showed divergent courses, suggesting roles for genetic or environmental modifiers. While features like craniosynostosis and mental retardation were noted, the review did not report dysmorphic findings such as telecanthus or brachydactyly, highlighting the need for more comprehensive phenotypic documentation in cases of NAH. Absence of overt thyrotoxicosis during initial years of life, as well as the presence of peculiar phenotypic features, makes this case different from previously reported cases. 

Table 2 summarises the data from 19 previously described cases with a focus on initial presentation and distinctive features. Features suggestive of fetal thyrotoxicosis, of which prematurity (most common), poor weight gain, tachycardia, goitre, hepatomegaly, splenomegaly, accelerated bone maturation, craniosynostosis, stare, and eyelid retraction have been reported in both familial non-autoimmune congenital hyperthyroidism (FNAH) and SNAH. Several phenotypic presentations congruent to fetal thyrotoxicosis, such as motor and speed delay, facial hypoplasia, ventriculomegaly and scaphocephaly, cerebral palsy, supra-ventricular tachycardia, and mitral valve prolapse have been reported in cases of familial NAH. 

**Table 2:** Clinical characteristics, management, and outcomes in reported cases of sporadic non-immune hyperthyroidism Table 2 summarizes published case reports and series of sporadic non-autoimmune congenital hyperthyroidism (SNAH), detailing genetic mutations, perinatal characteristics, clinical features, and treatment outcomes. Mutations are listed as per reported nucleotide or amino acid substitutions. Extra-thyroidal features include craniofacial, neurodevelopmental, and skeletal anomalies. “N/A” denotes data not available in the original article. Reference numbers correspond to those cited in the main reference list [1,2,5-7,9,14-27]. HPE: Histopathological examination; MMI: Methimazole; PTU: Propylthiouracil; ATD: Antithyroid drug.

Sr. No.	TSHR mutation	Onset of hyperthyroidism	Age at diagnosis	Gestational age/Birth weight/Birth events	First presentation of goitre	Craniosynostosis/Birth defects/Additional Extra-thyroidal features	Thyroid storm	Radio-iodine received at the age of	Number of Relapses post ablation/medical therapy	Surgery/HPE	Reference
1.	Ser281Asn	Two months	Four months	34 weeks/2.35 kgs	Four months	Craniosynostosis, proptosis, goitre, midface hypoplasia, dolichocephaly, adenoid hypertrophy with re-growth of adenoids post surgery, obstructive sleep apnea, advanced bone age, hypotonia	Yes	N/A	N/A	Adenoidectomy and tonsillectomy; no thyroidectomy at the time of report	Chester et al. (2008) [14]
2.	Ser281Asn	At birth	Two days	38 weeks/2.41 kgs	N/A	Advanced bone age	Yes/ multiple relapses on MMI	No	N/A	Total thyroidectomy at age 6.9 years	Scagalia et al. (2012) [15]
3.	Met453Thr	At one month	Eight months	36 weeks/3.04 kg	No goitre at birth. Multinodular goitre at seven years	Severe asphyxia, Splenomegaly at birth. Exophthalmos at 8 months, recurrent respiratory tract infections, retarded psychomotor development. Advanced bone age (4 years at eight months)	No	9 years (persistent disease post subtotal thyroidectomy)	Three (received 4 ablations; 150, 150, 200 and 500 MBq; 9 to 13 years, hypothyroid at 16 years).	Sub-total thyroidectomy at age nine years. Follicular adenomas on HPE	Lavard et al. (1999) [16]
4.	Met453Thr	Antenatal	At birth	32.5 weeks/1.69 kg	At birth (diffuse goitre)	Marked ocular signs (stare, retraction, proptosis). Hepatic, splenic enlargement, thrombocytopenic purpura, jaundice, and small fontanells.	No	No	N/A	No surgery done at the time of the report.	de Roux et al. (1996) [17]
5.	Ser505Asn	Antenatal	Five months	38 weeks (vacuum)/ 2.6 kg	15 months	Advanced bone age, failure to thrive, mild exophthalmos, microcephaly, speech delay	No	N/A	One relapse post medical therapy	Near total thyroidectomy at 27 months	Hozapfel et al. (1997) [6]
6.	Leu512Gln	10 days of life	Neonatal (10 days)	32 weeks/ 1.86 kg	No goitre at birth, large goitre by adulthood (370 ml at age 20)	Craniosynostosis (surgery at 1.5 years), perodactyly, hydrocephalus, advanced bone age, mild mental retardation	No	Yes (at 20 years)	N/A	No surgery (thyroidectomy was recommended, but declined)	Nishihara et al. (2006) [18]
7.	Ile568Thr	At birth	15 days	35 weeks/2.05 kg/ Normal vaginal delivery (uneventful except GDM in mother)	At birth	Advanced bone age, staring/lid retraction, mild dyslalia	No	N/A	Recurrence on ATD tapering	No surgery at time of report; predicted need for near-total thyroidectomy	Tonacchera et al. (2000) [19]
8.	Val597Leu	Nine months	Nine months	37 weeks/ 2.5 kg	Slightly enlarged on CT scan	Advanced skeletal maturation (bone age 4y2m at 10 months)	No	N/A	Persistent thyrotoxicosis despite antithyroid therapy	Yes (total thyroidectomy at 14 months)	Esapa et al. (1999) [20]
9.	Phe631Leu	At birth	At birth	32 weeks/1.66 kg	At birth (diffuse goitre)	Advanced bone age (early years), frontal bossing, mental retardation (IQ 75 - 85), hyperactivity	No	9.2 years	Multiple relapses on therapy, persistence after subtotal thyroidectomy	Yes (subtotal thyroidectomy at 8.7 years; hyperplasia with multiple nodules)	Kopp et al. (1995) [5]
10.	Ile568Thr	Fetal	Day 9	35 weeks/ 2.55 kg, Spontaneous vaginal delivery	No goitre	Meconium-stained at birth, tachycardia, hepatomegaly with transaminitis. Failure to thrive. Advanced bone age. Supraventricular tachycardia at 9 months	Yes (First month of life)	No	N/A	Not planned while reporting	Watkins et al. [21]
11.	Thr632Ile	Neonatal	At birth	33 weeks/ 1.45 kg/twin delivery (normal euthyroid twin brother)	Not palpable at birth but confirmed at surgery	Premature sagittal suture synostosis, neurologic impairment, mild mental retardation (IQ-62)	No	Planned at age 19	Relapsed post near-total thyroidectomy, regrowth of remnant, recurrence by age 14 and 19	Yes (Near total thyroidectomy at 3 years, nodular hyperplasia), remnant excised at 14 years	Kopp et al. (1997)[22]
12.	Asp633Tyr	45 days	Six months	36 weeks/ 2.4 kg/ Vaginal delivery	Six months (Grade I, confirmed on scinitiscan)	Craniosynostosis, dolicocephaly, hypertelorism, highly arched palate, archnodactyly, marfanoid habitus	No	No (planned but not done during follow-up)	Multiple relapses after three surgeries and multiple ATD courses.	Yes (3 subtotal/ near-total thyroidectomy at 10, 13.1 and 20.2 years, persistent hyperfunction post-op with nodular transformation	Bircan et al. (2008) [1]
13.	Ser281Asp	First few weeks of life	Four months	36 weeks/ 2.52 kg	No enlargement clinically	Craniosynostosis (surgery at one year), staring eyes, elevated blood pressure	No	No	Multiple relapses on ATD	Sub-total thyroidectomy done at six years; no further relapses noted post-op	Gruters et al. (1998) [7]
14.	Ala623Val	Neonatal	Six months	39 weeks/ 3.5 kg	Six months	Advanced bone age (Three years at six months), no craniosynostosis, normal development	No	No	One relapse occurred when antithyroid treatment was stopped at 12 months	Surgery planned; not done at the time of the report	Aycan et al. (2010) [23]
15.	Ala428Val	Two weeks	Two weeks	37 weeks/ 2.55 kg/ Vaginal delivery	At 4.6 years (on US)	Apnea, hyperactivity	No	No	No relapses, hyperthyroidism controlled with carbimazole for 5.9 years	No surgery as of report time	Borgel et al. (2005) [24]
16.	Ser505Asn	Neonatal	Post-natal, Exact age not specified	38 weeks / 2.6 kg/ Vacuum extraction	No goitre initially, thyroid enlargement during antithyroid therapy	Mild exophthalmos, very small anterior fontanelle, persistent low head circumference, psychomotor and speed delay, bone age four to six years at six months	No	No	Recurrence on ATD dose tapering	Thyroidectomy done at 27 months	Schwab et al. (1996)[25]
17.	Pro639Ser	Around 20-24 months (based on rising growth velocity)	24 months	40 weeks/ 4.0 kg	No goitre (normal gland size on US throughout follow-up)	None reported	No	No	Methimazole was started at 30 months and continued effectively	No	Agretti et al. (2012) [2]
18.	Asp633Glu	Fetal	24 days	33 weeks/ 2.28 kg/Emergency cesarean section	Detected on US and Tc-99m scan shortly after birth	Grade 3 Intraventricular hemorrhage, ventriculomegaly, hydrocephalus (VP shunt), craniosynostosis, delayed milestones, advanced bone age (2 yrs 8 months at 12 months), bladder stone, urinary tract infection, precocious puberty, mild intellectual disability (IQ-65) [26]	Yes (treated with PTU and propranolol)	No	Relapsed on tapering methimazole	No surgery done (managed medically as of till report)	Cho et al. (2018) [9]
19.	Val656Phe	Fetal	7th day	38 weeks/3.11kg/Normal vaginal delivery	Goitre (diffuse thyroid hyperplasia) at birth	Mild hypertrophy of the left ventricle. (Early suspicion and initiation of treatment prevented most neonatal manifestations)	No Received Methimazole and propranolol at day 7	No	Persistent requirement of ATD to maintain euthyroid states	No surgery planned as of the ast follow-up at the 25th month	Kayas et al. (2022) [27]
20.	Asp633Glu (Present case)	Fetal	Seven years (No thyrotoxic features till age 7y)	preterm (34 weeks + 2 days, vaginal delivery Birth weight 2400 grams.	Grade 1 goitre at presentation at seven years. No goitre at birth	Delayed developmental milestones (more affected in cognitive and fine motor domains, while preserved gross motor and social domains). Unique dysmorphic features: Brachydactyly in all 4 limbs, Ocular telecanthus, Flat nasal bridge, Mitral valve prolapse	No carbimazole since age seven years. Persistently increasing dose.	15 years	One. Received 2nd RAI ablation after six months.	No surgery planned. Post-ablative hypothyroidism.	Not applicable

Moon Bae Ahn revisited the Korean case initially reported in 2018 with a focus on extra-thyroidal manifestations, where ventriculomegaly, vesicoureteral reflux leading to pyelonephritis were important observations [26]. However, mitral valve prolapse and short 3rd/4th/5th metacarpals (Figures 1B, 1E) as well as brachymetatarsia, a condition with congenital short third and fourth metatarsals (Figures 1C, 1F), have never been reported in sporadic NAH. The presence of hypertelorism, flat nasal bridge, and low-set ears is also a new finding in our case (Figures 1A, 1D). 

Amongst previously described cases of SNAH, with few exceptions, almost all had overt thyrotoxicosis either during the neonatal period or infancy. Recent systematic reviews of germline TSH receptor activating mutation-associated hyperthyroidism, encompassing both familial (FNAH) and sporadic (SNAH) cases, highlight marked variability in age of onset and clinical severity. Infantile-onset disease, most often sporadic, tends to present with severe thyrotoxicosis and intrauterine features, whereas childhood-onset cases are predominantly familial and usually manifest with overt but less severe hyperthyroidism. Adult-onset disease is largely familial and may present with milder or even subclinical forms [28]. On the contrary to this, though our case was preterm with low birth weight, features of overt hyperthyroidism in the form of mild autonomic overactivity were significantly delayed as compared to previously reported cases. Unlike most cases of sporadic nonimmune hyperthyroidism, which were severe since first presentation, our case during the initial three years after diagnosis had controlled activity, but remained uncontrolled throughout the subsequent five years of follow-up despite a high dose of antithyroid drugs. 

Most of the previously described cases had some degree of mental retardation or developmental delay, which is a consistent finding in our case. However, other prominent findings like craniosynostosis, hydrocephalus was absent in our case. Due to the rarity of the case, treatment modality is not definitive, and various combinations have been described in the literature. In view of severe disease, radioiodine ablation with or without thyroidectomy remains the only option to arrest persistent hyperthyroidism with frequent relapses on medical therapy.

## Conclusions

In summary, we report the first documented case of sporadic nonautoimmune congenital hyperthyroidism (SNAH) from the Indian population, caused by an activating TSH receptor mutation: Asp633Glu with a novel pathogenic variant (c.1899C>G). To the best of our knowledge, this is the first reported case globally of sporadic non-autoimmune congenital hyperthyroidism (SNAH) presenting with distinctive dysmorphic features, including bilateral brachydactyly involving the third to fifth metacarpals in all four limbs and ocular telecanthus. The patient presented with delayed onset, mild disease activity at initial evaluation, and extrathyroidal phenotypic features, which collectively obscured early diagnosis. Given the persistent hyperthyroidism and progressive disease course despite prolonged medical therapy, earlier consideration of definitive thyroid ablation might have improved long-term outcomes in this case. However, due to the rarity of SNAH and limited literature guiding optimal timing of intervention, therapeutic decisions currently remain individualized and empiric. This case underscores the importance of maintaining a high index of suspicion for SNAH not only in neonates and infants, but also in older children presenting with persistent thyrotoxicosis in the absence of thyroid autoantibodies. Early recognition and timely intervention may prevent irreversible complications such as intellectual disability and improve long-term outcomes.
